# A case study of group art therapy using digital media for adolescents with intellectual disabilities

**DOI:** 10.3389/fpsyt.2023.1172079

**Published:** 2023-05-02

**Authors:** Jinkyung Kim, Yeo Ju Chung

**Affiliations:** Cha University, Seongnam, Gyeonggi, South Korea

**Keywords:** group art therapy, digital media, intellectual disabilities, adolescents, case study

## Abstract

**Introduction:**

In art therapy, digital art therapy is a new method in which clients use digital media to express themselves creatively. We wanted to explore what this means for adolescents with disabilities. The purpose of this qualitative case study was to explain what kind of experience they had when digital media was applied as an expressive and therapeutic medium in group art therapy in which adolescents with intellectual disabilities were participants and what kind of therapeutic meaning the experience had. We tried to know the therapeutic factors by extracting the implications of meaning.

**Methods:**

Participants were second-year high school students with intellectual disabilities who belonged to special classes. They were selected in an intentional purposive sampling method. Five teenagers with intellectual disabilities participated in 11 group art therapy sessions. Data were collected through interviews, observation, and digital artwork collection. Collected data were case studies analyzed using an inductive approach. In this study, the use of digital media was defined and utilized as “Digital Art Therapy” by setting the scope related to the study according to client’s behavioral method.

**Results:**

As a digital generation accustomed to smartphones, the participants gained confidence by repeatedly acquiring new technologies through familiarity with the media. Interaction with the media through touch and the use of apps have promoted autonomy with interest and pleasure to disabled teenagers, allowing them to express themselves actively. In particular, digital art therapy induces a holistic sensory experience by mobilizing visual images that could represent various expressions, emotions felt in music and tactile senses that made texts for people with intellectual disabilities with difficulty in verbal communication.

**Discussion:**

Art therapy using digital media has become an important experience that provides opportunities to arouse curiosity, enjoy creative activities, and express positive emotions vividly to adolescents with intellectual disabilities who have difficulties with expression and communication and a sense of lethargy. Therefore, it is suggested that an in-depth understanding of the characteristics and differences between traditional and digital media is necessary, and that complementary use to help create therapeutic purposes and art therapy is important.

## 1. Introduction

We are currently living in an era of digital transformation. The emergence of the 4th Industrial Revolution has led to an acceleration of digital technology. The COVID-19 pandemic has only further accelerated the use of digital technology (1). New digital technologies including metaverse, Augmented Reality (AR), and Internet of Things (IoT) focusing on artificial intelligence (AI). Telemedicine and online education are all expected to spread, evolve, and continue to be extensively developed with increasing demands throughout the world (2, 3).

One arena in which the importance of digital technology can be highlighted is art therapy. Art therapy can promote self-exploration and understanding by using the creative process of artwork as a means of self-expression and communication (4–6). Recently, the field of art therapy is using online platforms to activate community and creativity among therapists (7), With the COVID-19 pandemic started in 2020, the field of art therapy is rapidly transitioning to remote work to cope with the pandemic (8). It also prepares for the future by improving accessibility and acceptability to treatment using new art media (9).

Digital media was first used as a communication tool in art therapy by Weinberg (10), who attempted to apply computer technology to rehabilitation for people with disabilities. The medium used in art therapy is a media of expression. Digital media has been attracting attention as a new tool to attract interest and provide opportunities for artistic expression due to the characteristics of digital media (11).

In the new media era, digital media has evolved and developed in a wide range of areas, including remote art therapy, phototherapy, animation, and virtual reality (VR) using computer and graphic programs as well as creative expressions (12, 13). Recently, numerous apps for digital art production have been released that target a wide range of users, including many young customers of the digital generation. Despite the explosive spread of art-making apps (e.g., apps for digital drawing and painting, visual journalism, storytelling, animation, and multimedia), many art therapists lack experience and skills in using digital media in treatment (14). Moreover, research on this topic has progressed relatively slowly (9). Those who were born in the digital age, i.e., “digital natives” (15), recognize the utility of technology in treatment with familiarity and convenience (16). They also prefer to express their feelings through images, text, or video in their work activities, as they consider the digital space their main space (17, 18).

Malchiodi (11) has stated that digital media accessible to people with disabilities can promote a new therapeutic relationship. Previous studies have shown that the use of digital media with hospitalized children (19, 20) or in limited medical environments in clinical art therapy can allow patients to virtually experience media that they cannot experience in real life (11). Studies have revealed the usefulness of digital media in art therapy because digital media can enable patients to experience media that they could not experience in real life through digital media (21–24).

International Classification of Health of Functioning Disability was approved by the World Health Organization (WHO) in 2001. International Classification of Functioning, Disability and Health (ICF) defines “disability” as an interaction between multidimensional factors at the individual, social, and physical levels (25). People with disabilities have more limited access to digital technology than the general public (26, 27). The National Telecommunications and Information Administration (NTIA) has reported that people with disabilities have digital information gaps in their online activities and internet use in various environments compared to people without disabilities (28). In the rapidly changing environment of the modern world, digital technology’s hyperconnectivity, convenience, and subjectivity are more needed for people with disabilities who require some form of assistance or have physical constraints. To reduce such digital information gap, there is a need for digital inclusion that does not exclude or marginalize anybody (29). Moreover, using digital media for communication is essential for students with disabilities to successfully transition to society after graduation and live meaningfully (30).

According to the US National Center for Education Statistics (31), from 2020 to 2021, 7.2 million students from 3 to 21 years old received special education services under the Disabled Education Act (IDEA), thus accounting for 15% of all public school students. This number has also been showing an increasing trend. Intellectual disability is defined as a condition characterized by significant limitations in intellectual functioning and adaptive behavior before age 22 (32, 33).

According to the American Psychiatry Association (APA, 2013), intellectual disability is a condition that begins during development and is characterized by deficits in both intellectual intelligence and adaptive functioning in conceptual, social, and practical domains (34). In the Republic of Korea, intellectual disabilities account for the most significant proportion of the total number of students with disabilities at 51.8% (35). For teenagers with intellectual disabilities, attending special classes can cause various psychological stresses such as anxiety and perceived inferiority because of one’s disability, the formation of low self-esteem, frustration, and atrophy due to frequent failure experiences (36). They might also face difficulties in social adaptability due to emotional expression that is either socially marginalized or limited and contraction of interpersonal relationships (32). Therefore, there is a greater need for therapeutic intervention for psychological and emotional problems of students with intellectual disabilities than among general students.

Art therapy for adolescents with intellectual disabilities can provide them opportunities for expression and communication through alternative visual media (37). Art therapy in the educational scene was, initiated by Edith Kramer (38), It has been shown that art could be a powerful communication tool based on all humans’ creativity (39). In addition, for students with disabilities who are usually protected and raised, art provides them a significant opportunity to function independently. They can feel joy, pleasure, and a sense of achievement in the experience of controlling materials at will (40).

In the Republic of Korea, art therapy services are provided during creative experience classes and after-school classes in special schools and general schools for children and adolescents with disabilities. For children and adolescents with special needs, art therapy in a school environment can provide them an opportunity to overcome obstacles to educational success (41, 42). In addition, strengthening programs for students who are identified as disabled can help them realize their social and academic potential by promoting appropriate social behavior and emotional development (41, 42). In this way, the clinical work of art therapy is gradually expanding into education and special education environments (43).

Technology provides many people with pleasant experiences and new opportunities (44). The playful interaction behavior offered by digital media provides sensory enjoyment, which drives art creation (45–48). The spontaneous and action-promoting characteristics of such interaction behavior have positive functions that can be useful for adolescents with intellectual disabilities who have low expression, who are depressed, who have low self-esteem, and who lack attention and motivation (49). Studies on the use of digital media for people with intellectual disabilities in art therapy (49–51) are very lacking, especially in group art therapy for adolescents with intellectual disabilities. It was difficult to find case studies that applied it as a medium of communication. Therefore, in art therapy using digital media, there is a need for extensive research on various subjects and case studies to understand the therapeutic process.

This study proposes a new method of participation for creative expression, selection, and control by therapeutically applying digital media, a form of communication and self-expression culture, to teenagers with intellectual disabilities so that they can enjoy the universal right to human equality. Moreover, a case study has been performed to obtain specific knowledge by understanding and exploring research participants through a therapeutic process and to understand the phenomenon in the art therapy process. To this end, the following research questions are explored in this study:

1. What are experiences of adolescents with intellectual disabilities in group art therapy using digital media?
2. What are meanings that can be extracted from experiences of adolescents with intellectual disabilities with group art therapy using digital media?
3. What are therapeutic factors of group art therapy using digital media for adolescents with intellectual disabilities?

## 2. Materials and methods

### 2.1. Research design

The present work used a qualitative case study approach to explore and understand experiences of individuals who had group art therapy in 11 sessions, from surface level observations in a natural setting to in-depth analyses (52). Phenomena of specific cases were described in a deep and natural context. New meanings were discovered based on the experiences reported by participants and data obtained from the phenomena observed by the researcher (53). The goal of the case study method was to investigate how, why, what was happening and what factors were related to the phenomenon (54). The purpose of qualitative case study is to explore and understand the experiences, meanings, and therapeutic factors of research participants through various data collection and analysis steps, while considering characteristics of manners (55).

### 2.2. Research procedure

The participants in this study were five students (one male student and four female students) in special classes at M high school in S city. The group art therapy program was conducted once a week from September 2019 to December 2019 by connecting creative experience classes for a semester over a total of 11 sessions (120 min for each session). This study used an institutional-centered art therapy program composed for special purposes. After explaining the purpose and method of the study to the teacher in charge of the special class, this study was reviewed and approved by the school.

Participants were all second-year high school students with intellectual disabilities who belonged to special classes. They were selected with a purposive sampling method (52). Based on the selection criteria for adolescents who submitted written consent, research participants and their parents (main guardians) were explained about the purpose and procedure of this study.

Personal information and characteristics of the participants in this study were obtained based on reports of special class teachers and observations of researchers. In this paper, aliases were used to protect research subjects (Table 1).

**Table 1:** Personal information as well as social and emotional characteristics of research participants.

Participants	Diagnosis	Personal information, social and emotional characteristics
Pink (17 years old, female)	Mild intellectual disability	She looks sensitive to her skinny appearance with short hair and glasses. Her learning ability is at the level of sixth grade in elementary school. Her learning ability is the best among the five students in the second grade of special classes of the high school. Both of her parents work and live with their sisters, brothers, and grandmothers. However, her parents have a low degree of interest in her, making it difficult for her to manage personal hygiene. Due to a lack of counseling for her worries or a lack of an ability to express her thoughts and feelings, she tends to not easily talk about stress or problems. She just accumulates them inside. She is blunt and passive. She shows dependence on her friends.
Summer (17 years old, female)	Mild intellectual disability	She looks tall and healthy. She has tied up long hair and wears round glasses. She smiles well when she makes eye contact. She uses her left hand. Her learning ability is at the level of fourth grade in elementary school. However, her sociality is the highest among the included students. Her mother and older brother also have intellectual disabilities. Thus, she feels that there are no one at home who can consult her concerns or understand her. She has the most active personality, although she cannot express her feelings well. She tends to be obsessed with people who understand and care for her. She likes to receive attention from others. She tends to make her friends take her side. She is popular with her friends for her smile and outgoing tendencies.
Handsome man (17 years old, male)	Mild intellectual disability	His first impression was that he had a healthy-looking body and was neatly dressed. Although he has a learning ability at the level of fifth grade in an elementary school, he does not show a willingness to do everything. Until elementary school, he studied in general school. However, In middle school, he was transferred to special classes, and in high school he was diagnosed with an intellectual disability, including Asperger’s Syndrome, and was registered as disabled. As an only child, he is under a lot of stress due to the clash between the differing educational views of his father and mother. He has violent emotions because he spends a lot of time at home playing games and watching TV. His parents’ interest level in him is high. However, his family’s guidance regarding personal hygiene management, time concepts, and regular lifestyle habits is insufficient for him. He is the only male student in the class who makes no eye contact. He typically keeps his head bowed. He makes few verbal expressions. He looks passive and lethargic.
Winter (17 years old, female)	Mild intellectual disability	She has short hair and a cute appearance. She has a learning ability at the level of third grade in an elementary school. She experiences a lot of stress about community life because she lives in a temporary shelter for youth. She tends to try to reach out to others first and wants to be loved by others. She is very stubborn about what she wants to do. She is active in forming ties with others. She has good friendships.
Sea (17 years old, female)	Mild intellectual disability	She has long hair with a tall and feminine appearance. She makes no facial expressions, as she simply answers questions. She has a learning ability at the level of fourth grade in an elementary school. She has good sociality, but she tends to only maintain relationships with people she likes. She is interested in makeup and fashion. She is an only child. She has a deep attachment to her mother. She wants to get other people’s attention. However, she does not express or approach first. Therefore, she experiences difficulties in forming friendships. She is firm due to her stubbornness. She does not express her feelings well. She has severe mood swings. Currently, she has little interaction with her classmates, and has distant relationships with those around her.

### 2.3. Case conceptualization

A case conceptualization is a method and clinical strategy for organizing obtained information about clients, for understanding and explaining clients, planning treatment interventions, and for establishing a consistent treatment strategy (56, 57). Case conceptualization is essential in psychotherapy (58). It was developed for art therapist responsibility and confidence in treatment (59). It has been used to increase the likelihood of treatment goals (57). Thus, the researcher identified problems, causes, and strengths based on various information related to the main complaints of participants and planned treatment using case conceptualization to obtain further improved results (Table 2) (60, 61).

**Table 2:** Case conceptualization.

Main component	Case concept explanation
Background and referral information	This case considered five intellectually disabled adolescents in the second grade of a special class at a general high school, and the therapeutic issue was commissioned by a special class teacher due to emotional difficulties and conflicts with peers.
Strengths	Willingness to participate in art therapy, supportive families and schools, the degree of intellectual disability was not severe, and accessibility allowing for the quick establishment of an alliance with the media without resistance to digital media.
Limitations	First, difficulty in expressing emotions, and second, lack of interpersonal skills.
Diagnostic impressions, Treatment guidance, prognosis	Due to the abundance of visual opportunities, digital media is essential for people with intellectual disabilities who do not understand the language well.
	Therapeutic digital media that affects emotional and emotional changes are used to express and communicate art therapy to promote various types of emotional expression.
	To improve the sociality of participants through group interactive art therapy that fosters collaboration using small social groups for treatment purposes (60).
	In the treatment process, the difficulty level was applied, specifically, step by step; considering the characteristics of adolescents with intellectual disabilities, techniques and materials were repeatedly utilized, and play elements were applied (61).
	The strategy was to have participants share symbolic images, strengthen interactions, and experience a sense of accomplishment and confidence.

### 2.4. Data collection and data analysis

Data were collected through interviews, observation, and document collection using the triangulation method (62). The average interview was about 30 min, there were two semi-structured interviews after initial and mid-term sessions along with one group interview after the end of sessions. Group dynamics were observed by having participants fill out semi-structured questionnaires to obtain abundant information (63).

Before starting the interview, the researcher explained to the participant that the interview content would be recorded and transcribed by the researcher, with anonymity and confidentiality of the interview content guaranteed. Consent was then obtained from each participant before the interview. We also shared individual results with participants after the study. In this process, terms related to informed consent were converted into easy-to-understand terms according to the level of understanding of the participants. The researcher proceeded with open questions within a large framework of the research problem (63). Participants were able to understand the purpose of this study and express their opinions based on their own experiences.

The document collection process included audio recordings and transcriptions of each session of art therapy by researcher, observation notes taken on-site, structured behavioral evaluations, and texts and artworks (audio-visual materials such as images and videos). Data analysis involved describing cases and contexts. Repeated comparative analyses using open coding, categorization, and category verification procedures were performed (64).

The researcher tried to find meaningful patterns, concepts, and topics by repeatedly reading the data (65). The interpretation summarized greater meanings of cases discovered by the researcher through analysis strategies using direct interpretation from the overall perspective according to the researcher’s intuition, reflective thinking, use of imagery, and application and search of new and diverse perspectives (66).

### 2.5. Trustworthiness

To secure the reliability of this study, opinions were exchanged with an academic adviser during research analysis and interpretation. The appropriateness of the data collection and coding contents was supervised. The researcher focused on revealing the experience and meaning of the research participants through data analyzed with a focus on fairness and consistency to minimize preconceptions. In order to exclude the researcher’s subjectivity as much as possible and to strengthen the sensitivity and reliability of the collected data, colleagues with a master’s degree who participated as observers and assistant therapists checked before and after the session and received feedback. A peer debriefing session was then held (67).

### 2.6. Ethical considerations

This study was conducted in class hours and within the scope of the curriculum at the special class of the school. After explaining the purpose and intended use of the study to all participants and their guardians in advance, written consent was voluntarily provided by them. The written consent form promised to protect the information and ensure anonymity of the participants. Participants and their guardians were explained that research data of this study would be discarded after being stored in a safe place for 5 years.

Ethical problems related to the use of digital media were supplemented by considering ethics codes for the confidentiality of electronic information suggested by the British Columbia Art Therapy Association (5), the American Art Therapy Qualification Board (68), and the Korean Art Therapy Association (KATA) (69).

All six iPads used in this study were set and encrypted with the researcher’s IDs so that information on these six iPads used in this study would not be leaked. After using the app, the tablet device used by participants was initialized to prevent data leakage. All data files stored in the form of electronic information were stored with passwords. All recorded contents and data were guaranteed to be discarded following completion of the study.

### 2.7. Procedure: description of group process

**Figure 1:**
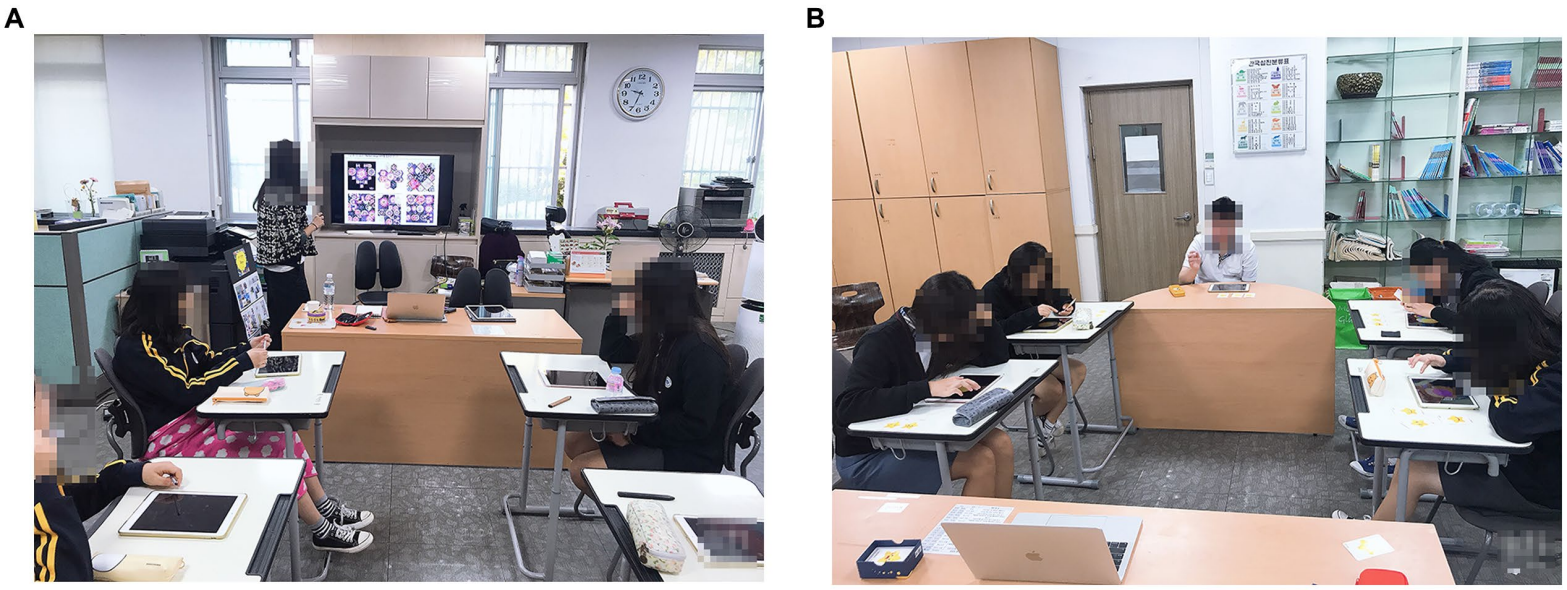
**(A)** Sharing of audio-visual materials. **(B)** 1:1 iPad art therapy environment.

In the art therapy environment, students’ desks were relocated to the center to face each other, and their interactions were observed. Technical education and practice on the iPad and the app are known to be important (70). Thus, a TV monitor located in front of the classroom was connected to the researcher’s laptop and iPad to model the app’s usage and iPad drawing process. Audio-visual materials on art therapy topics were shared by participants (Figure 1A). Moreover, five new iPad 9.7 32G units were rented for participants to have a 1:1 iPad art therapy environment (Figure 1B).

### 2.8. Treatment plans

This study applied Richter’s (71) “Art therapy’s approach to art therapy education based on education” by changing the difficulty and technique of digital media in consideration of the characteristics and learning levels of adolescents with intellectual disabilities (72).

The goal of art therapy is to improve emotional expression and strengthen sociality so that participants can complete high-quality works through the complementary usage of traditional media and digital media (73). Participants planned to freely express their psychological state using various functions of media as a generation familiar with digital media (74, 75). When using digital media in art activities, the therapist’s competence and ethical understanding of the media are essential factors (22, 76). The researcher participated in Today at Apple (experiential group session) provided by the Apple Store to increase their literacy and acquire skills in digital media utilization. They also received training on how to use apps, devices, photos, videos, coding, art, and design. The researcher demonstrated and supplemented a direct program before each session. The therapy was based on the experience of using digital media in clinical practice.

The researcher chose an iPad and iOS app considering that they are familiar to teenagers using smartphones, easy to access, and easy to carry. Specific expression techniques used in digital art therapy include digital painting, photography, video, stop motion, and digital collage. Apps used in this study for this are summarized in Table 3.

**Table 3:** Apps and functions used in digital art therapy.

Used session	App	Functions
2	Storymation Studio: Disney Edition (2019)	An app that uses snapshots to create a stop-motion movie. It can add screen effects to a photo image and insert music.
3, 4, 6	Procreate	An app for sketching, painting, and illustration. More than 135 brush libraries, layers, and time-lapses are available.
5	Perfect Image	A photo editing app that even beginners can use easily. Collage, Add Text, Magic Brush, Filter, Frame, and Sticker Features are available.
7, 8, 11	Clips	A free app to create and share videos. It has filters, moving text, music, emoticons, Disney character stickers, and short story add-ons.
9	Stop Motion Studio	An app to make a stop-motion movie. It can perform photo capture with time difference, movie editing, image editor, and music insertion functions.
10	Line Camera	A photo editing app. It has a collage, sticker, filter function, and video shooting.
6,7,8,9, 10,11 For warm-up	Mooda	An emotion recording app. Users can easily select emotion icons, attach photos, and record in a simple diary format.
Complete sessions	Camera	Installed by default on smartphones. Photo shooting, slow motion, video, and panorama functions are available.
Complete sessions	AirDrop	A function to transfer content such as photos, videos, and documents from one device to another through Bluetooth, and that allows sharing with MacBooks, iPads, and iPhones.

Storymation Studio [Application software]. (Version 1.0.2) Disney Edition. Seattle, WA: Wonder Forge (2019); Procreate [Application software]. (Version 5.3.2) Cupertino, CA: Savage Interactive Pty Ltd.; Perfect Image [Application software]. (Version 5.0.5) TongShuo (2010–2022); Clips [Application software]. (Version 3.1.3) Cupertino, CA: Apple Distribution International (2017); Stop Motion Studio [Application software]. (Version 11.4.4) Bluffton, SC: CATEATER, LLC. (2010); Line Camera [Application software]. (Version 15.5.5) Tokyo: LINE Corporation (2013); Mooda: Seoul [Application software]. (Version 1.14.0) OLIVESTONE Corp. (2019); Camera [Application software]. (Version iOS 15) Cupertino, CA: Apple Distribution International (2021); AirDrop [Application software]. (Version iOS 7) Cupertino, CA: Apple Distribution International (2011).

### 2.9. Sessions

The digital art therapy program was conducted in three stages: initial, intermediate, and advanced (Table 4). It was structured in consideration of cognitive, social, emotional and short-term aspects of adolescents with intellectual disabilities. After the intermediate stage, it was flexibly changed and finally implemented so that participants could autonomously decide on the topic they wanted. In terms of digital media utilization, the process and changes in the session were observed in an initial session followed by ten further sessions. The first session used traditional media. Another five sessions (2, 5, 7, 9, 10) had the complementary use of traditional media and digital media. Another five sessions (3, 4, 6, 8, 11) used digital media.

**Table 4:** Step-by-step goals of the digital art therapy program.

Step	Treatment Goal
Initial stage (Sessions 1–3)	Relieve tension and build trust
	Arouse interest through the exploration of digital media
Intermediate stage (Sessions 4–8)	Self-exploration and emotional expression
	Interaction and communication
	Enhancing agency and autonomy
	Expansion of expression through digital media
Advanced stage (Sessions 9–11)	Positive emotional experience
	Increased self-confidence through a sense of accomplishment
	Improve social skills

#### 2.9.1. Initial stage (sessions 1–3)

In the initial stage, the focus was on relieving tension and building a relationship of trust. To build trust with participants, guidelines for art therapy and the rules to be followed within the therapy time were set together.

##### 2.9.1.1. Session 1: creating my own story

**Figure 2:**
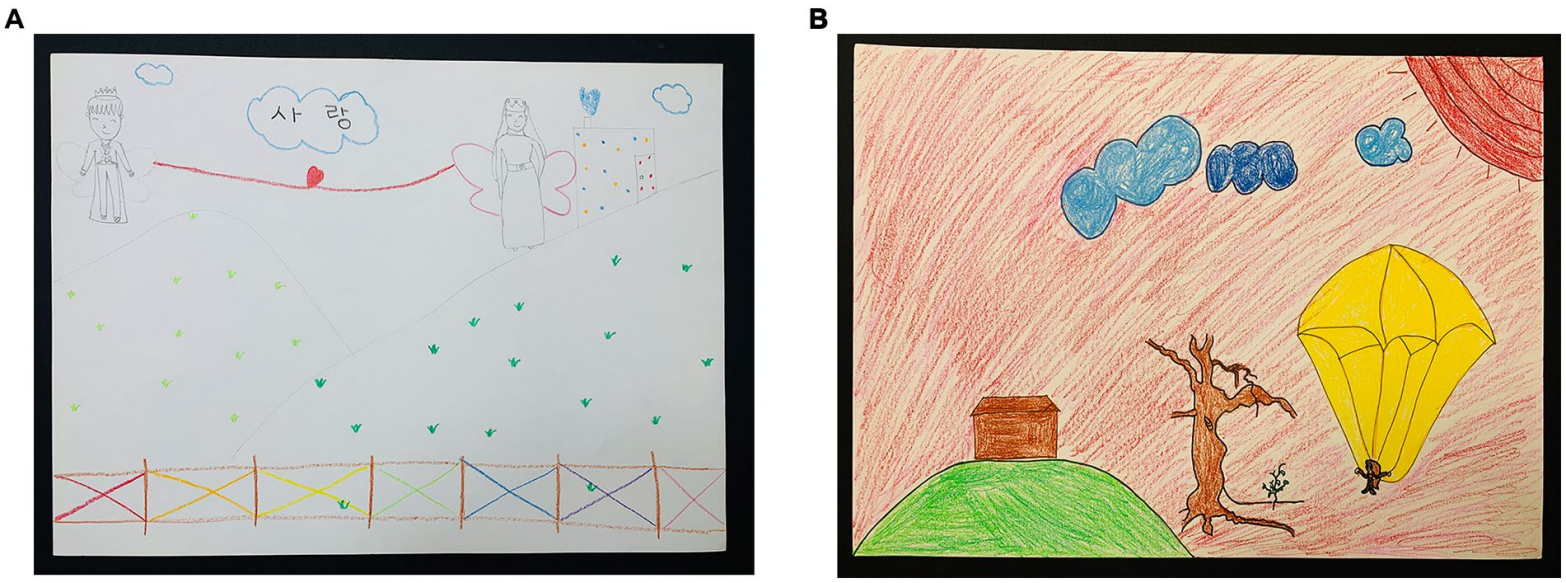
**(A)** Summer (Happy love). **(B)** Sea (Landscape).

In the first session, traditional media (Colored pencil, felt-tip pen, and eight-section drawing paper) and picture cards were used to draw story pictures (Figures 2A,B). Participants actively and sincerely engaged in the work process, although their progress was slow. For that reason, it seemed necessary to distribute planned time.

Participants were observed to habitually say negative words created by the experience of failure, such as “I’m doomed, “and “It is driving me crazy, “in the creative process. Thus, continuous emotional support and encouragement for the participants seemed necessary. The first session was a meaningful session as an opportunity for social interaction within the group, taking the first step in experiencing and expressing empathy by telling stories about themselves, although participants were not familiar with using art images.

##### 2.9.1.2. Session 2: safe space

**Figure 3:**
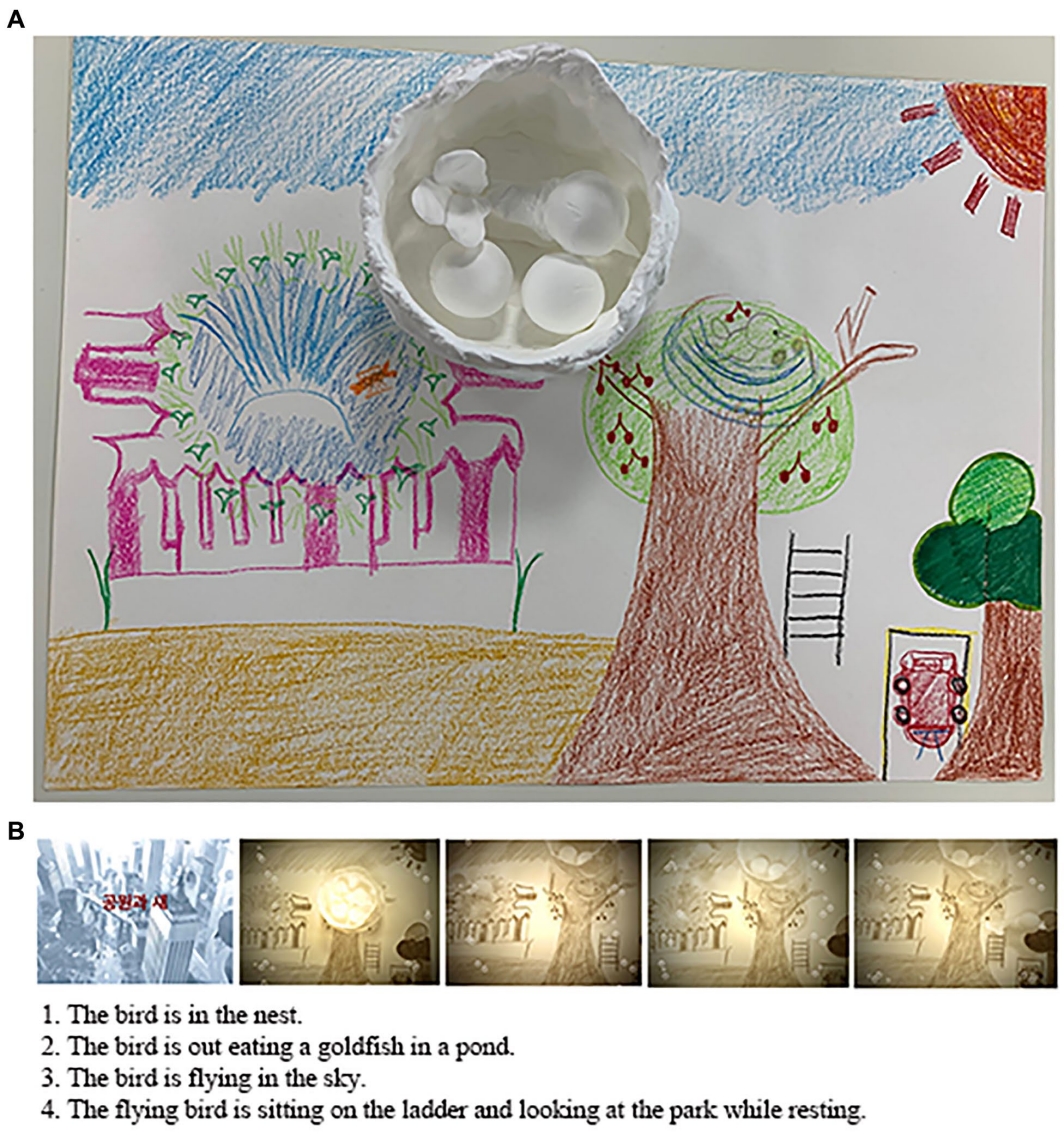
**(A)** Sea (Park and a Bird). **(B)** Sea’s stop-motion animation (see also Supplementary Video S1).

In the second session, clay was used for emotional expression or sensory activation (Figure 3A). Opportunities for expression were expanded by using different types of digital media together. The researcher used the easy-to-access Storymation Studio: Disney Edition app to motivate participants and attract the interest of teenagers who like animation. Participants intuitively searched through the media (77). They showed interest in the function of the app and willingness to use it on their own from filming to editing. In the process of creating an animation, participants said “Wow, it is like a game,” “It is like being a film director,” “It is fun,” “I like it because it is like a movie, “I like this music the most, and This is the prettiest, ‘unlike their relaxing appearance in clay work’”. They were careful in choosing backgrounds, effects, and music. Participants increased their interactions by showing their digital artwork (Figure 3B; Supplementary Video S1). While watching the completed artworks together, they were all delighted by the moving visual images and the vitality given to the animation by the music.

After the session, Sea approached the researcher and asked, “What’s the name of this app?” and said, “I want to go home and try it again” while smiling brightly. Participants showed emotional changes in the process of their artwork and put metaphors into the work. Attractive aspects of digital media also helped form a trust relationship between the participants and researchers.

##### 2.9.1.3. Session 3: creating emotions

**Figure 4:**
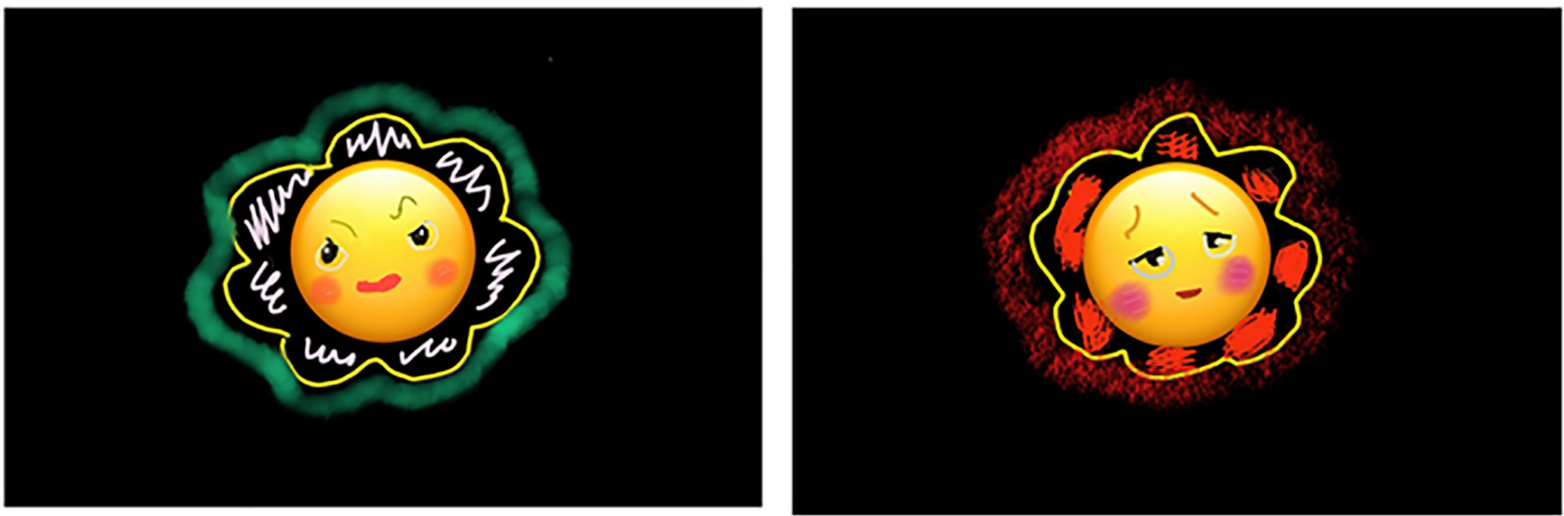
Winter’s emoticons “Unpleasant, tired” (iPad 9.7 inch-Digital drawing).

**Figure 5:**
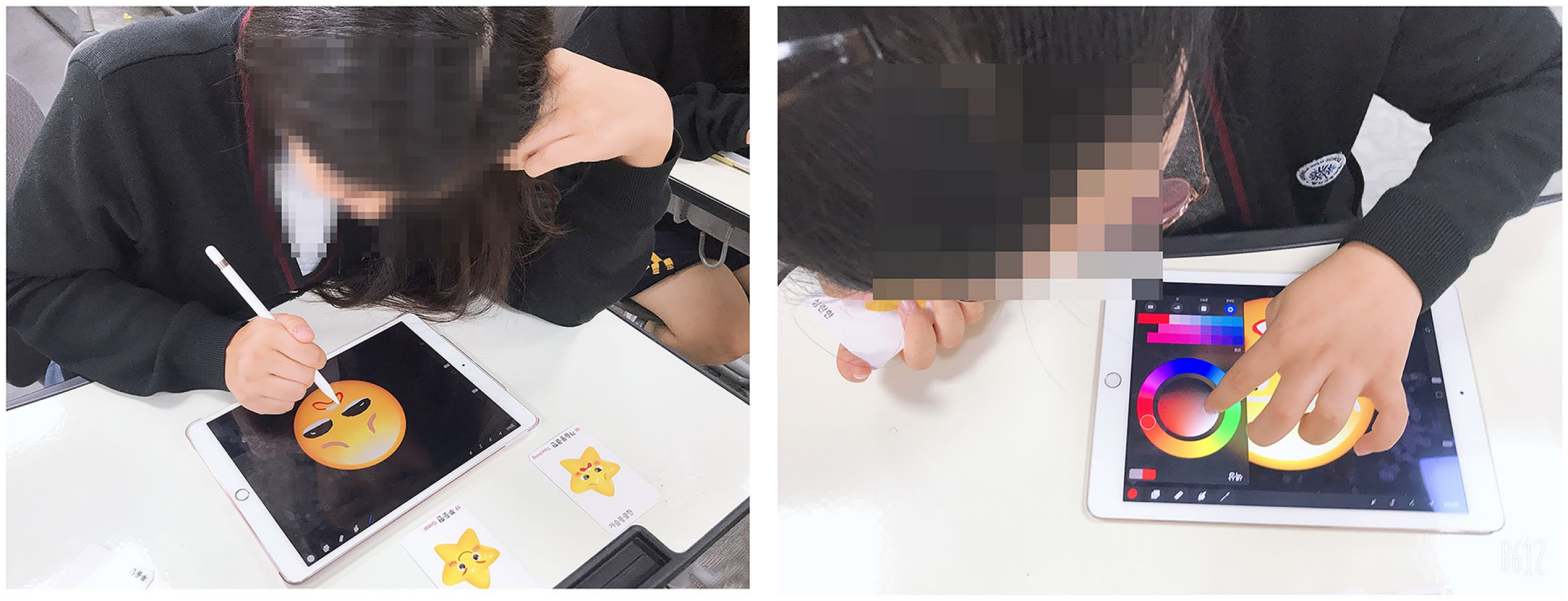
Process of iPad drawing (Apple pencil drawing and finger painting).

Before starting this session, the researcher modeled the process of drawing by connecting the iPad to a TV monitor. This session involved the use of the Procreate app under the theme of making friendly emoticons that could be easily accessed due to the role-played by smartphones in daily life (Figure 4). Participants used iPad as a canvas and worked as shown in (Figure 5) using their fingers and Apple pencil or Stylus pen.

This was an opportunity for participants to understand each other by sharing emotional images. After completion, they made statements such as “It was fun and fun after expressing my feelings,” “I felt relieved and comfortable,” and “I was just happy without thinking about anything.” All participants responded that it was much more fun than drawing on drawing paper. They were also satisfied with the convenience involved in resizing images using digital media and easily correcting mistakes.

Although it was difficult for participants to express emotions using spoken language, they could better express themselves through digital artworks. Participants were able to share information and understand each other by talking about emoticons that expressed each other’s feelings.

#### 2.9.2. Intermediate stage (sessions 4–8)

Starting from sessions in the intermediate stage, participants were given autonomy and flexibility so that they could decide on the desired topic on their own. The app was used repeatedly, so that participants would master the technical aspects.

##### 2.9.2.1. Session 4: a self-portrait of pop art

**Figure 6:**
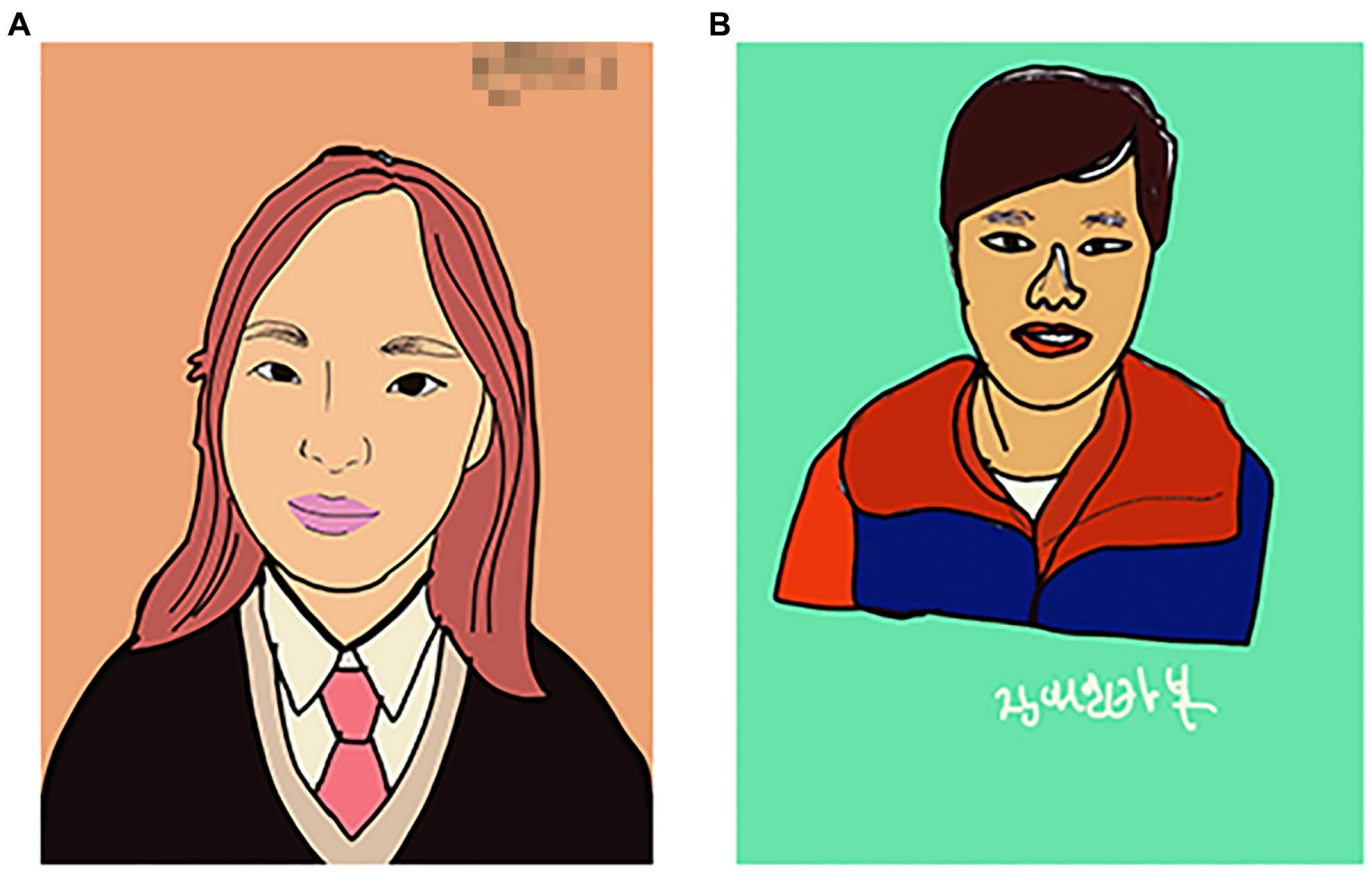
**(A)** Pink’s “Self-portrait”. **(B)** Handsome man’s “Disabled fool”.

In the 4th session, with the theme of self-portrait drawing in pop art, the Procreate app used last time was used repeatedly. Pink started by saying she was not confident, but after completing the self-portrait, Pink praised herself, saying that “I did a great job, Pink.” She was delighted to write her name on her work (Figure 6A). After completing the self-portrait, Handsome man wrote “disabled fool” on his work and showed a negative self-concept (Figure 6B). Handsome man recognized his disability and revealed his thoughts about himself through self-portraits.

Participants enjoyed the process of painting the self-portrait, expressed a sense of accomplishment after completion, and showed therapeutic importance of the artwork by valuing it. Participants familiarized themselves with technical aspects by repeatedly using the same app. During the talk time, they used the AirDrop app to instantly share their artwork which helped the group communicate.

##### 2.9.2.2. Session 5: me and our garden

**Figure 7:**
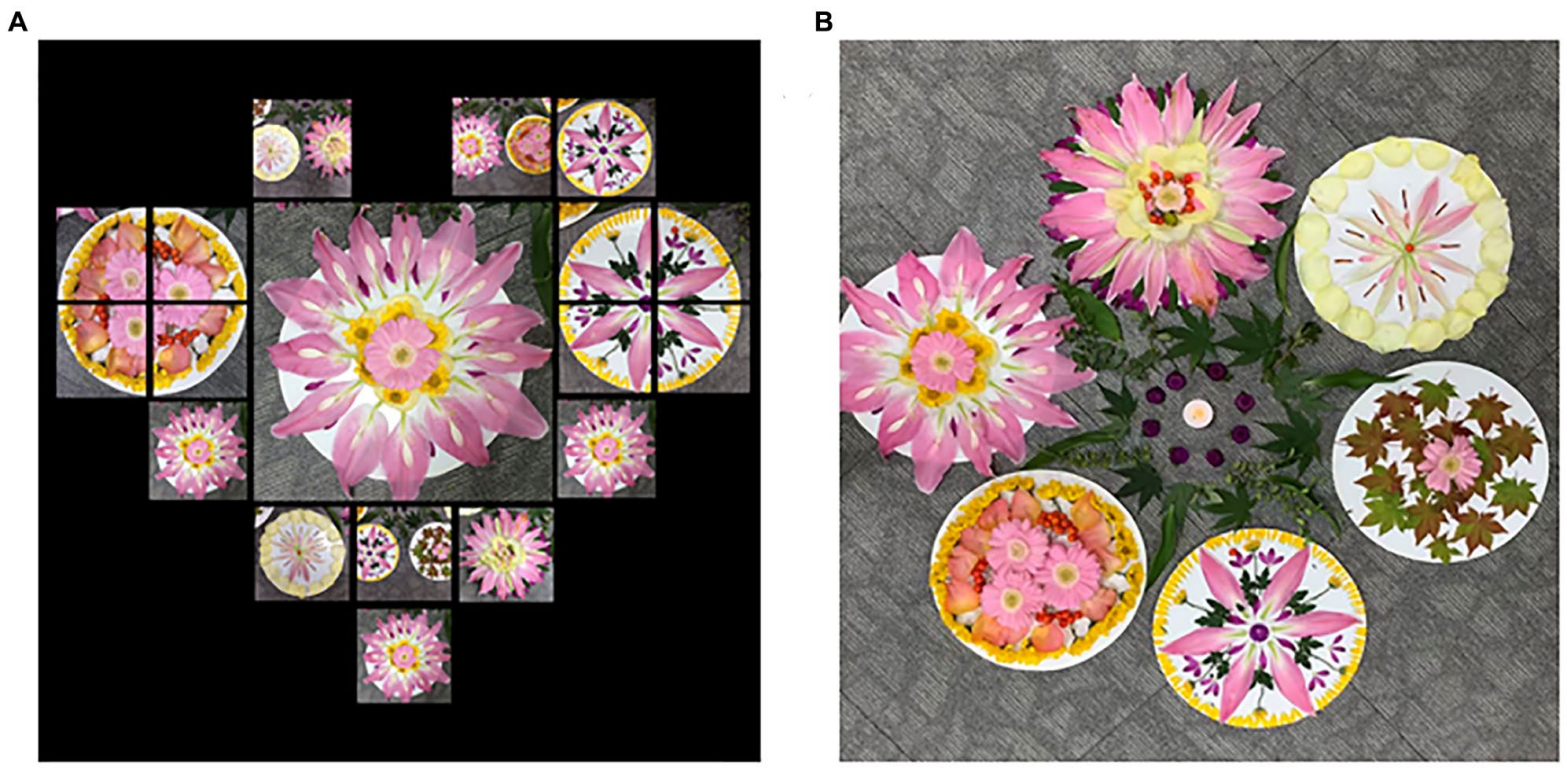
**(A)** Pink’s “Photo collage”. **(B)** Our garden (collaborative work).

In the fifth session, participants smelled the scent of flowers. The classroom was full of flowers, which awakened their senses, thus allowing them to have a moment of relaxation and healing. In this session, the Perfect Image app with a photo collage function was used. Participants all experienced unity through cooperative artwork while creating a garden together (Figure 7B). After completion, they captured their desired parts by taking pictures to recreate a photo collage (Figure 7A).

While participants were creating the mandala works (diameter 25 cm), their emotions were stabilized with positive emotions were aroused. There was a change in interaction that provided altruism, praise, and encouragement to friends. Handsome man took more than 140 pictures while producing a nice photo collage and said, “It’s a nice garden.” Actively created photos re-visualize images through photo collages and create new stories. Creative possibilities were also expanded to photo collage artworks that fused traditional media and digital media.

##### 2.9.2.3. Session 6: if I were ~

**Figure 8:**
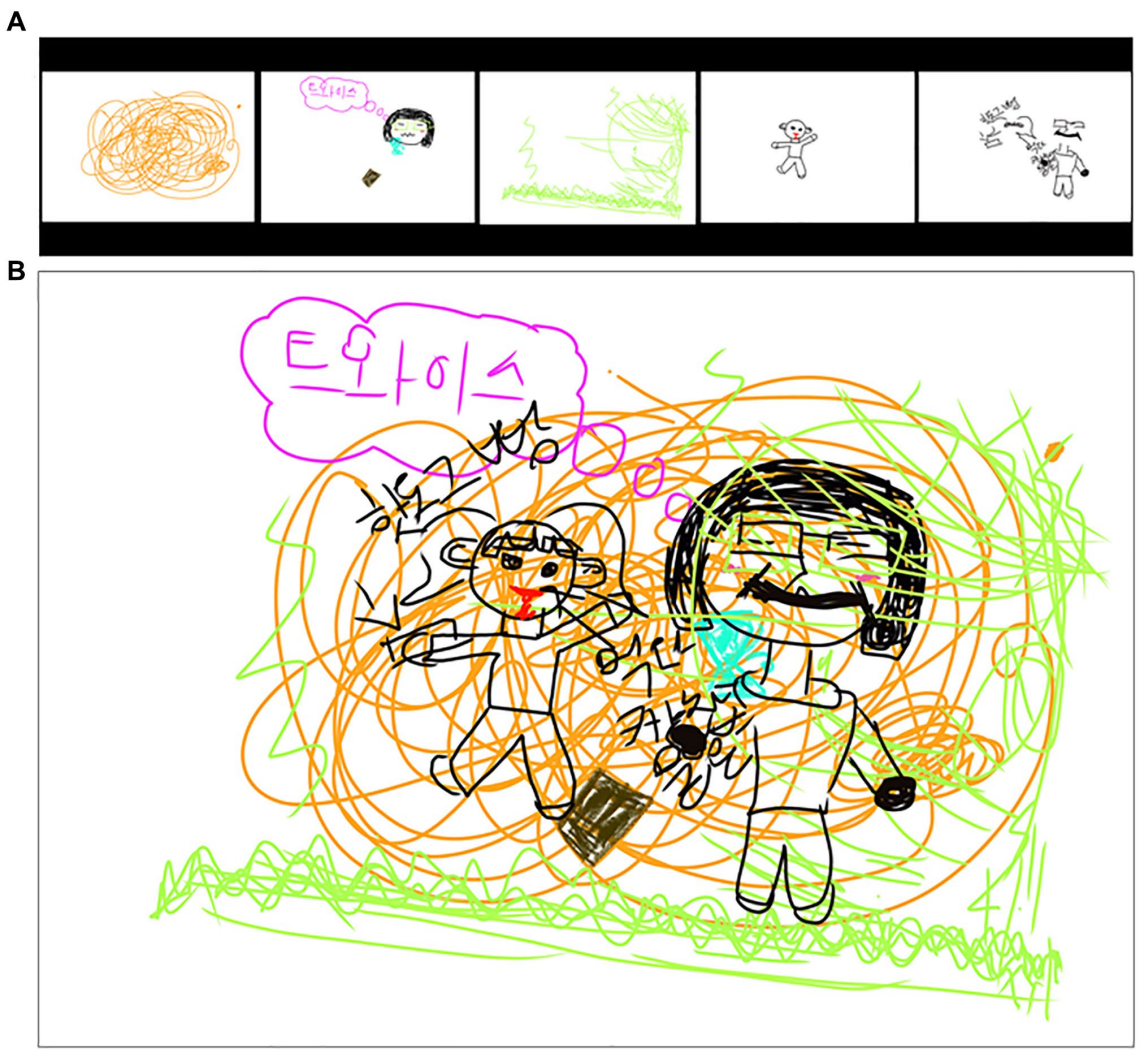
**(A)** Layer per participant. **(B)** Pink’s “I do not think anything”.

In the sixth session, the Procreate app was used. The participants drew pictures on their iPads, handed them over to other participants, and created a “Digital painting rotation” in which they drew pictures in addition to the pictures they received from other participants. Participants had to add layers to the received picture and draw it to prevent other people’s paintings from being erased, and the record of the picture was stored separately (Figure 8A). Therefore, it was possible to confirm the contribution of each participant to the single work.

Pink expressed her complex feelings with colors and lines, saying that she was not in good condition. When her painting (Figure 8B) was returned, she smiled, saying that she was pretty when she left, but that she was struck by lightning. Pink talked about the pleasant parts and the unpleasant parts in the additional painting. Digital painting, which can be drawn by dividing the picture into separate layers, was easy to modify later, and participants were able to engage in the activity with less burden.

This session gave participants opportunities to sympathize with others through perspective-taking, interest, and imagination. It was also an opportunity to express their future dreams and communicate with images.

##### 2.9.2.4. Session 7: making wishes

**Figure 9:**
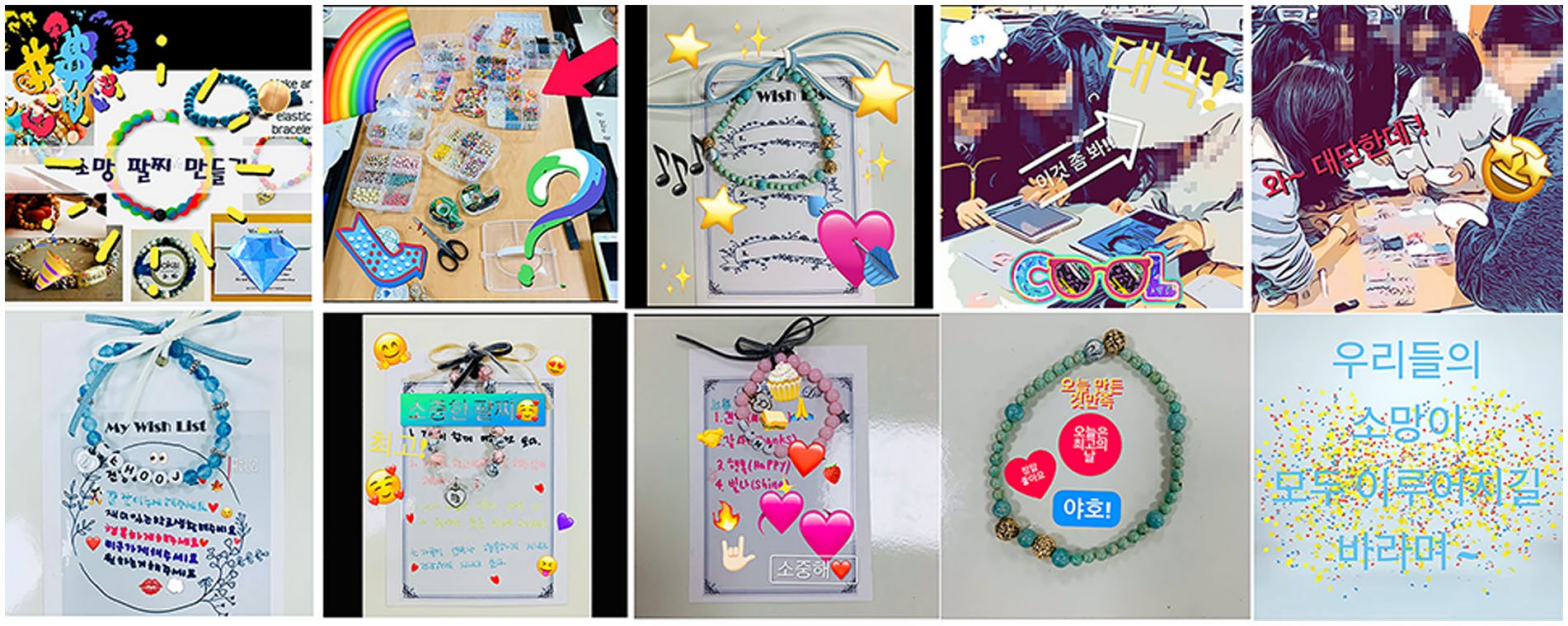
Wish bracelet video collaborative work. Soundtrack: Itsudemo by Asuka Ito (Genre: Bright fully) (The soundtrack is music that accompanies and synchronizes with the images of the participant’s video work and is edited by the participants directly selecting the music they want).

For the seventh session, Participants were motivated by the program of ‘Making a wish bracelet’ that they selected themselves. They worked with an immersive focus. This session involved creating a video (Figure 9) that documented the process of artistic expression as a Clips app for video production. The process of making bracelets gave them an opportunity to solve problems while being original. Participants chose colors, shapes, and materials. They edited videos together and expressed positive emotions through the experience of collaborative work.

Participants showed the importance of their digital artwork by inserting various moving stickers and text to express emotions. Sea expressed her joy and satisfaction by inserting the words. “Satisfaction with what I made today, Today is the best day, Hooray” in the video. Participants started to praise themselves by feeling a sense of accomplishment. They encouraged each other and showed positive relationship experiences and interactions in the group art therapy.

##### 2.9.2.5. Session 8: a fantasy trip

The theme of the trip, which was the most frequently expressed theme in participants’ wishes in the seventh session, was made into a video using the Clips app. Participants said they were happy just thinking about the trip plan. Photos of their travel destinations contained emotions and information through symbolic meanings. Participants were immersed in making videos of their dream trip. They expressed excitement and expectation.

**Figure 10:**
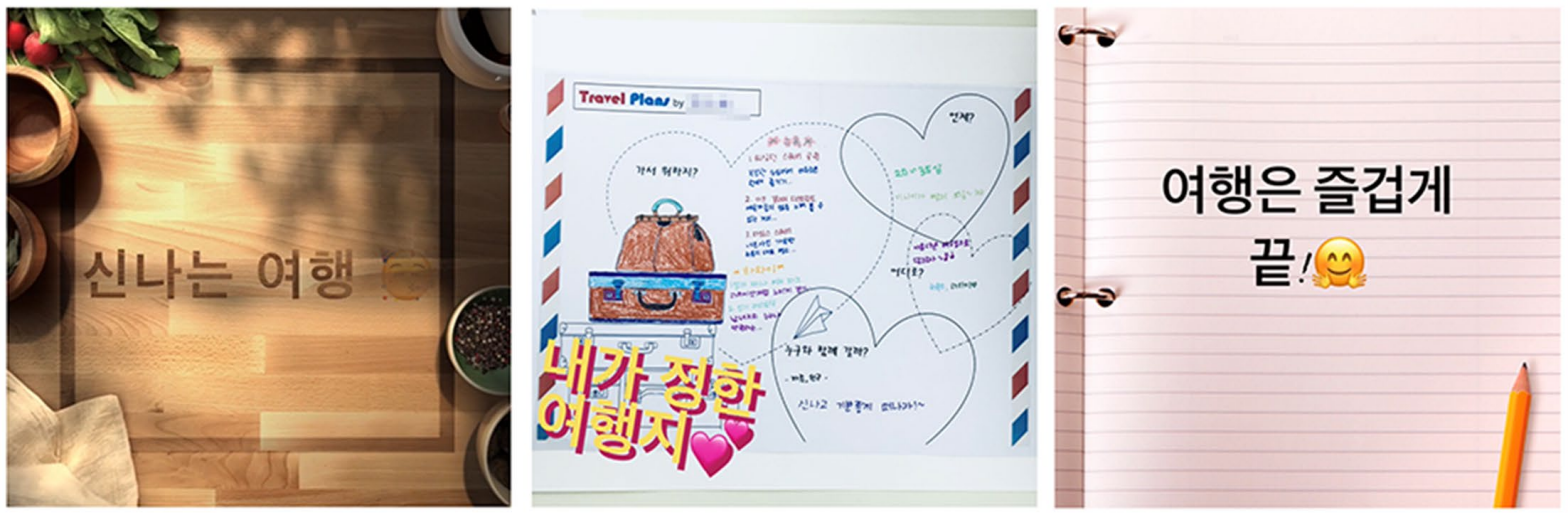
Summer’s “Exciting Travel” video. Soundtrack: So Young at Heart by Judson Crane (Genre: relaxed).

Summer often expressed her expectations and excitement after deciding on New York and Hawaii as travel destinations, for which she carefully planned, saying, “There are so many places I want to go here.” She made a highly complete work called “Exciting Travel” (Figure 10).

Participants were able to actively express themselves with audio-visual stimulation of multimedia. Emotions they felt in the music were harmonized with their visual stimuli. The liveliness of the completed video satisfied their emotions by mobilizing the touch of making text.

#### 2.9.3. Advanced stage (sessions 9–11)

In the advanced stage, with goals of enhancing self-confidence and improving social skills through positive emotional experiences and a sense of achievement, the process of change in the past was examined, focusing on completion of collaborative works, exhibitions of works, and art journaling.

##### 2.9.3.1. Session 9: our seasonal trees

**Figure 11:**
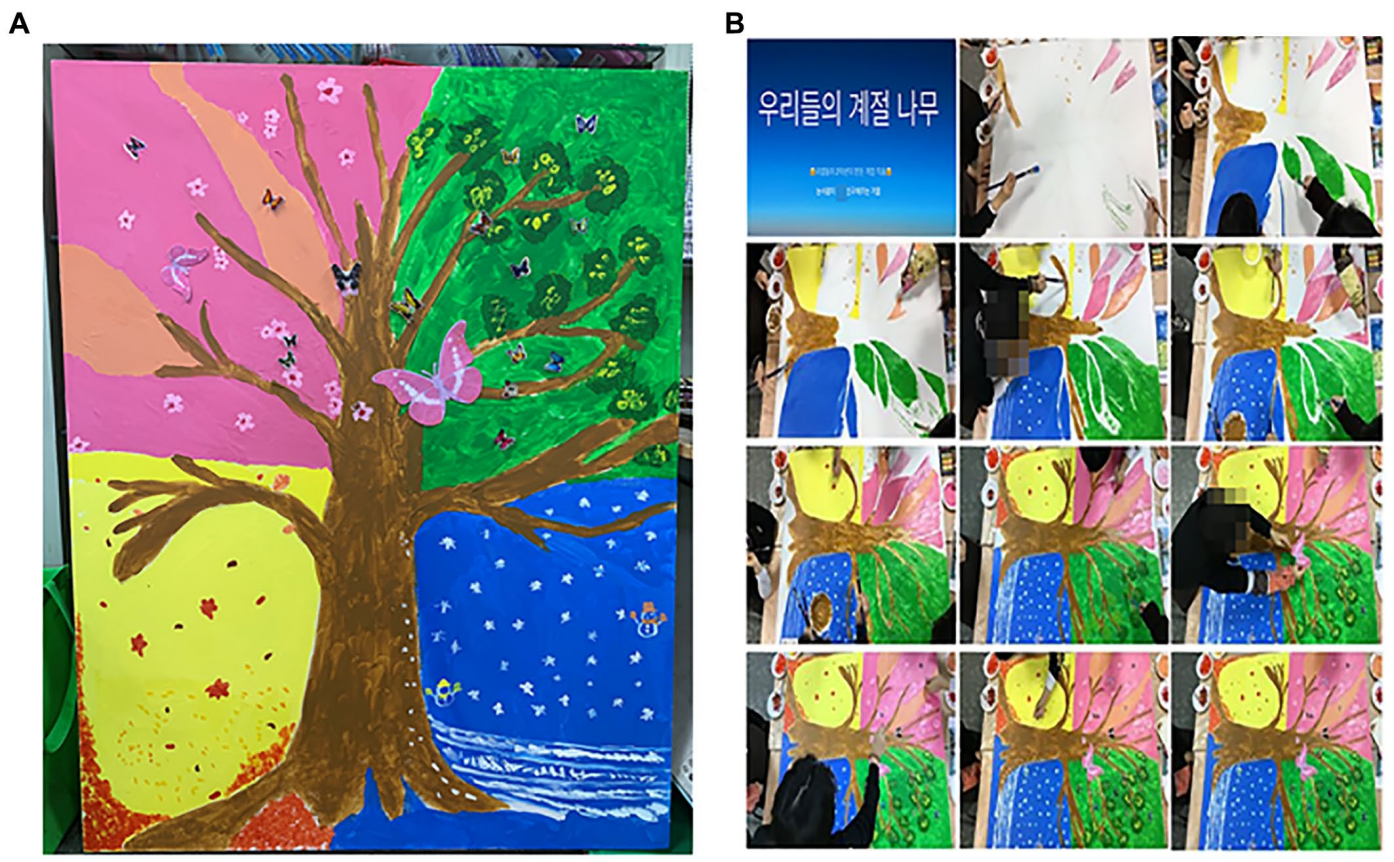
**(A)** Our seasonal tree (116.8 × 80.3) canvas, acrylic paint. **(B)** Stop-motion movie of our seasonal tree (Theme music: Cherry Monday) (The theme music is music that harmonizes with the image of the stop-motion movie work, and the members of the participants select and edit the music they want).

In the ninth session, the participants drew trees together on a 50P (Paysage) canvas as a collaboration project in the advanced stage. With the Stop Motion Studio app, the work activity process was captured with a total of 417 photos, ultimately leading to the completion of a 1-min and 25-s-long stop-motion movie (Figure 11B). Participants completed their respective areas, looked at the overall harmony, cared for and helped their friends, and showed a mature appearance that gave meaning to the importance of the process of completing the work together. Participants coordinated their opinions and showed teamwork in determining the title, music, background, and effect of the artwork together. They were also able to appreciate the stop-motion movie artwork and view their roles objectively. When setting the title of the work, Handsome man surprised everyone by loudly suggesting “Our Seasonal Tree” (Figure 11A).

##### 2.9.3.2. Session 10: Christmas gift

**Figure 12:**
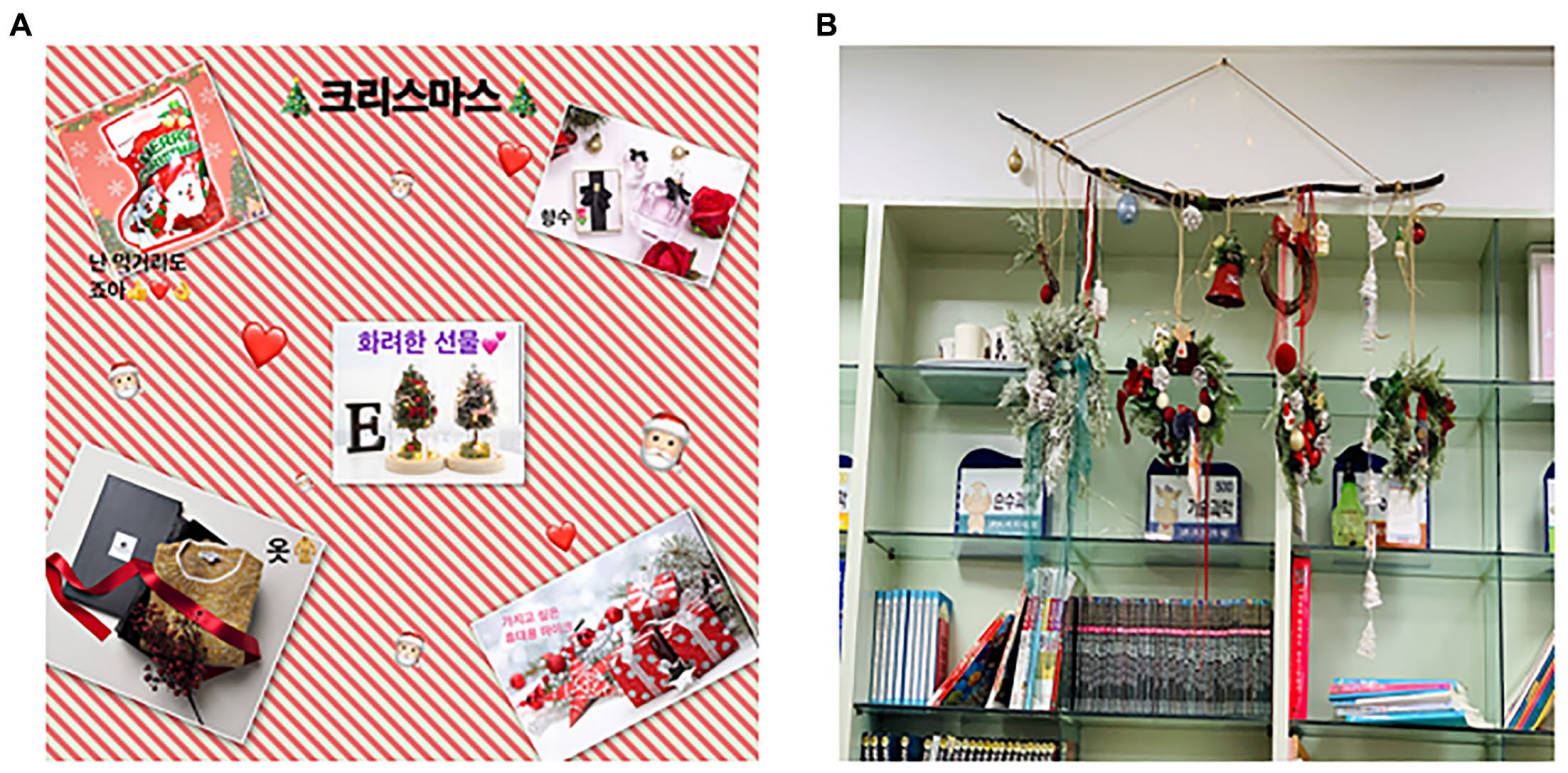
**(A)** Summer’s “Fancy gift”. **(B)** Collaborative work of Christmas garland.

This session began with a laugh among participants who prepared Santa hats and Rudolph headbands. The 10th session involves the creation of a digital collage (Figure 12A) and a Christmas wreath using the Line camera app. Participants had experience in magazine collage. Thus, they understood the digital collage quickly and completed the work in a short time because it was easy to edit. Parts that could be transformed without damaging the original, such as adjusting the size of the picture, aroused participants’ interest. Collage is a technique with the greatest metaphorical potential (78). Participants felt grateful and happy. They expressed positive emotions as they thought about the grand prize under the theme of Christmas gifts. Afterward, they gathered their completed wreaths and made a Christmas Garland together, turned on the lights, and cheered and applauded. Participants were very proud of their work. They took pictures together in front of their works (Figure 12B) and hung them in the classroom to display them.

##### 2.9.3.3. Session 11: say “goodbye”

As the “Media Art Exhibition” was held as a closing session that involved a group celebration, participants aimed to reflect on the process of growth throughout the therapy process and became aware of their changes. The 11th session involved using the Clips app to complete a modern media art journal in which images, narratives, music, and editing were done within a single digital media. During digital artwork activities, participants approached their friends and worked together. They showed an improved relationship wherein they were interested in. They cheered for their friends’ artwork. Titles of art journals created by participants reflected main themes of their experiences.

**Figure 13:**
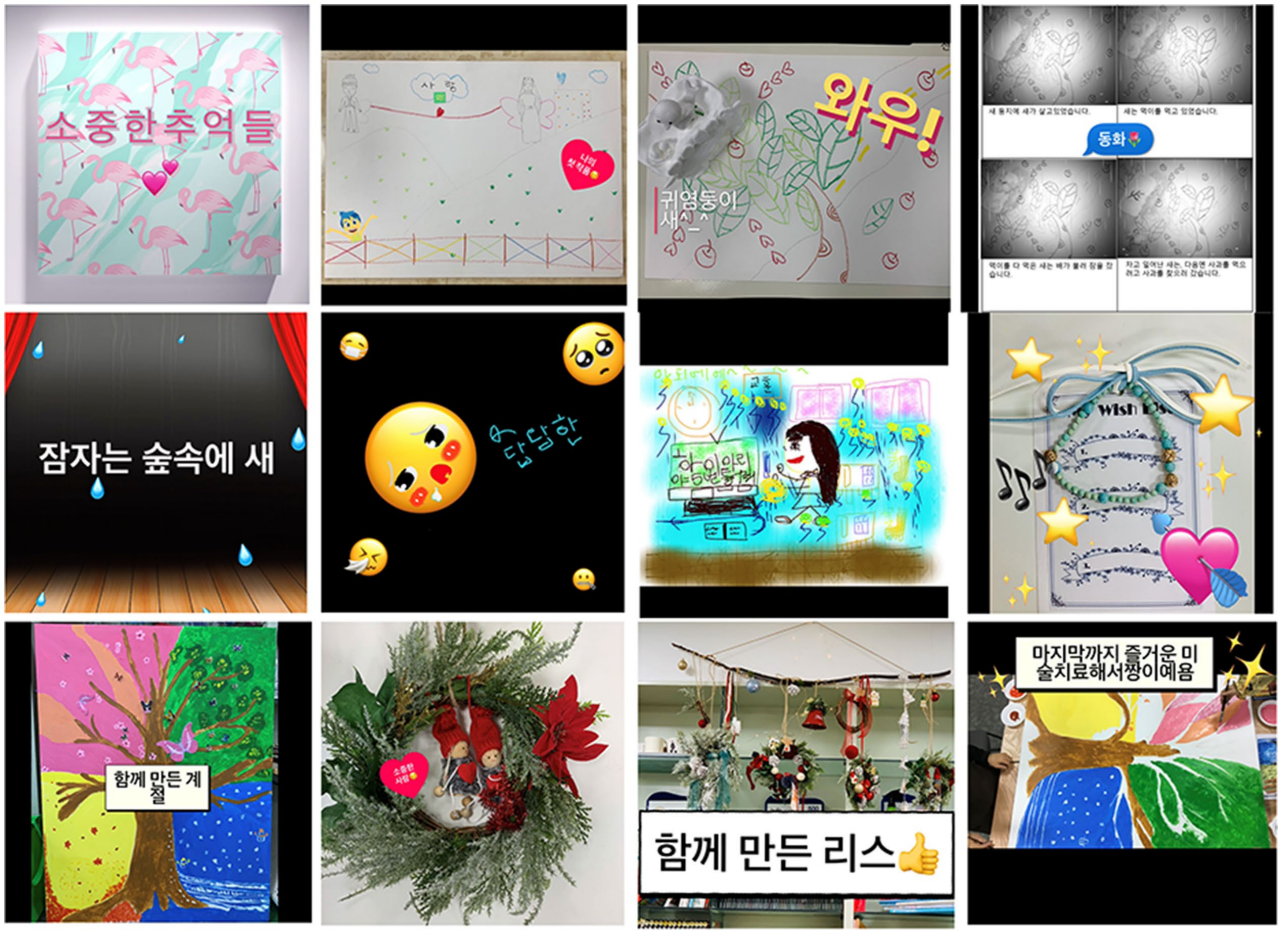
Summer’s “Precious memories” (Media art journal video). Soundtrack: Itsudemo by Asuka Ito (Genre: delightfully).

Pink was embarrassed while looking at her works in each session and danced while choosing music during the media art journal. She showed elevated expressions in terms of behavior as well as verbal expressions. Summer said that she could get to know more about her friends by watching the exhibition. She laughed and said about her work, “It’s a very well-made video, but there are so many things I want to say right now, so it’s a pity that I could not put them all into the work.” Images and writings had been added to each work to the end. Expressing high satisfaction after completion, the title of the artwork was called ‘Precious Memories’ (Figure 13).

## 3. Results

The main results of this study were an analysis of participants’ experiences and meanings of participants using digital media and descriptions of therapeutic factors.

### 3.1. Analysis of experience and meaning of group art therapy using digital media

Experiences and associated meaning of the group art therapy were identified through coding, categorization, and subjectification by comparing and reviewing artworks, interview transcription contents, and observation journals for 11 sessions of group art therapy. Meaning was derived from a total of six themes and 13 subcategories representing experiences derived through repetitive comparison and line-by-line analysis were found (Table 5).

**Table 5:** Results of group art therapy experience analysis using digital media.

Themes (6)	Categories (13)	Examples of quotations
New and fun digital art therapy	A new digital art therapy	“I’m excited, I am upbeat.”
		“It is fun to make videos.”
	Interest and fun through the act of play (art)	“Everything was new while I was in the art therapy.”
	Liveliness and enjoyment of digital artworks	“It was really fun and exciting, so I want to do it again.”
Enjoying daily choices as the protagonist of life	Experience choice through interaction with digital media	“It was good to be able to do it my way.”
	Convenience and subjectivity of digital media	“It is more satisfying and fun to do it myself.”
Discovery of the positive power and possibilities	A valuable experience improving the quality of life	“I want to do it again.”
		“I will try again by remembering various things I learned during art therapy, Thank you, iPad.”
	Digital art therapy that one wishes to experience again	“It is mine, a really well-made video.”
Digital art therapy enriching experience	Expanded representation and total sensibility through digital media	“Thank you so much for a fun and precious experience.”
		“It was difficult to express in words, but expressing it through the app made me feel very refreshed.”
	Natural convergence of multimedia and various emotional experiences	“It was a good memory because I did a lot of artworks through art therapy.”
Preciousness and joyfulness of the process of “being together”	Communication and connectivity through digital media	“There were parts where I thought the same and parts where I did not, but it was still fun.”
		“There were times when it was hard, but it was good to contribute to a part of the collaboration.”
	Expansion of relationships through collective collaboration	“It was a little uncomfortable and complicated to cooperate together, but I was proud of it after completing it.”
Sublimation to digital art through immersion	Creation process and immersion of digital art	“Teacher, I’m showing the greatest concentration in my life!”
	Digital media environment, a space for appreciation and expression	“Why does time pass so quickly?”

#### 3.1.1. Theme 1. New and fun digital art therapy

In the interview, participants said that their first experience of creating work using an iPad was “incredible.” This new experience might represent the first step toward life improvement and change.

Participants had more stories and laughter as they created artworks with digital media. Interactions with digital media can enhance the aspect of play. Their effects give pleasure (79). Fun is the core of humanity (80). Fun has important implications for human development (81).

#### 3.1.2. Theme 2. Enjoying daily choices as the protagonist of life

Participants felt more freedom and satisfaction in the session about subject they voluntarily selected. Adolescents with intellectual disabilities have limited choices and opportunities due to family control and social exclusion, and they experience exclusion due to a lack of awareness regarding technology and digital information (82). Digital media works cannot be created without active participation (45, 83). Since its use itself represented a choice, it was confirmed that it was meaningful for participants to enjoy daily options as a protagonist of life.

#### 3.1.3. Theme 3. Discovery of the positive power and possibilities

Participants said that their experiences in art therapy would be helpful in their daily lives. These responses mean that they discovered their hidden potential through digital art therapy and felt positive power such as competence and confidence. Participants were more motivated by the acquisition of skills in the use of iPads and apps.

#### 3.1.4. Theme 4. Digital art therapy enriching experience

Rubin (38) has stated that adolescents with intellectual disabilities have a greater desire for emotional experiences and creative activities due to various deficiencies. Participants experienced various new self-expressions and rich emotional experiences through convergence between media. The difference between digital art therapy and traditional art therapy is that digital media has a non-material aspect, which not only amplifies bodily sensations but also triggers heightened behaviors, bringing participants another bodily experience.

#### 3.1.5. Theme 5. Preciousness and joyfulness of the process of “being together”

Participants said that the experience of group art therapy would represent a pleasant memory. They put the importance on cherishing times during which they complete the work with friends and collaborated happily together. Through group art therapy, participants attached meaning to the importance of collaboration. The connectivity of digital media helped communicate by allowing their work to be shared immediately and quickly. It also improved sociality by having a positive effect as a tool for communication between participants, thus improving their social skills.

#### 3.1.6. Theme 6. Sublimation to digital art through immersion

Participants said that when making digital art works with iPads, they concentrated better, and time went by faster. This meant that digital media increased participants’ immersion and motivation to participate (84–88) and sublimated them into emotional sublimation through the expression of catharsis and integrated digital art creation in the art therapy process.

### 3.2. Therapeutic factors of group art therapy using digital media in adolescents with intellectual disabilities

Therapeutic factors that were extracted and used to investigate the meaning of the group art therapy experience of adolescents with intellectual disabilities using digital media were playfulness, interactivity, and scalability of digital media (Table 6) (9, 48, 89–97).

**Table 6:** Therapeutic factors of group art therapy using digital media.

Therapeutic factors	Description of therapeutic factors
Playfulness	The playfulness of digital media (89, 90) is a powerful therapeutic element. Various play factors, such as realistic images, colors, sounds, and touches, stimulated the curiosity and interest of intellectually disabled adolescents who had attention problems and synchronization defects. Therefore, it is a therapeutic factor because it has aspects of motivating (90) treatment, relieving tension, and expressing itself (91).
Interactivity	Interaction is the most important characteristic of digital media (92). Media and humans are influenced by interaction (93). In particular, playful interactions with digital media are attractive to children and adolescents (46). Creativity is created through immediate interactions with digital media, and the sensory space that is the result of interaction with media exists within the expressive characteristics of digital technology (94). Digital art therapy can comprehensively present visual images, colors, sound, photo editing, and storytelling, thus inducing a holistic sensory experience different from traditional art therapy (48). It can be used as a therapeutic factor by conveying strong meanings and emotions (95).
Scalability	The scalability of digital media led to the expansion of time and space, the expansion of expression methods, the expansion of physical constraints, the expansion of senses, the expansion of media, and the expansion of experience as therapeutic factors. In McLuhan (96)’s core proposition, the term of ‘media is human expansion’ means that the body and senses are expanded through technology. It is much more suitable as a medium that can expand creativity and flexibility for coping in a changing world (97). It can extend physical constraints to new environments that require high levels of participation from people with disabilities or underprivileged people. The use of digital technology in art therapy means new possibilities for extending adaptation and esthetic experience to new media beyond the increase in access to expanding time and space (9).

## 4. Discussion

This study aimed to explore participants’ experiences and meanings when applying using digital hardware tablets and software apps as delivery devices for expressive and therapeutic media in group art therapy for adolescents with intellectual disabilities.

Results of the present study are consistent with those of Weinberg (10), who argues that computer-based art therapy has unique advantages to grant curiosity and motivation for patients with disabilities as a new approach to a successful art experience. Malchiodi (11) has also shown that digital media can improve concentration and self-esteem, consistent with the results of the present study. This study is also in line with the study of Garner (12), who argues that the use of digital art media can improve choice and autonomy among students with disabilities. Wehmeyer and Metzler (98) have emphasized the importance of autonomy for students with disabilities to have a successful transition to social life.

Participants in the present work experienced sensory expansion, various emotional experiences, and natural convergence between media in the process of engaging in the artwork activities. This supports Mcniff’s belief (13) that all-in-one digital devices are promising as a fusion art production tool. The results of this study are also consistent with Hallas and Cleave’s (51) claim that, for many people, technology can increase opportunities for realizable experiences such as playing, creativity, and improving quality of life, therefore contributing to primary prevention and health promotion.

Therapeutic use of digital media can enhance the emotions of adolescents with less expression and lethargy. It can also maximize the experience of participants in various expression methods to provide satisfaction and achievement. Therefore, it has the potential to become a strong therapeutic factor. Characteristics of digital media also support studies of Ehinger (99) and L’Esperance (100), who have explained that digital media can increase adaptation and increase both communication and relationship formation in art therapy. Results of this study are also consistent with a recent study by Albrecht et al. (45), which has shown that interactive digital art can be a useful therapeutic intervention, particularly for people with intellectual and developmental disabilities.

This study was meaningful in that it applied therapeutic digital media to adolescents with intellectual disabilities in special education classes who had emotional difficulties to help them understand the process, and draw conclusions through an actual case analysis depicting the phenomenon of digital art therapy with rich and detailed technology.

## 5. Limitations and recommendations for future studies

First, this study considered adolescents with intellectual disabilities attending special classes in a high school in the Republic of Korea. Therefore, there is a need for future studies using various media to determine the possibility of using digital art therapy for adolescents with other types of disabilities.

Second, the initial purchase price of digital media is high. There might be an economic burden when it is used in group art therapy. However, in the long term, compared to high-quality traditional art materials, apps—which are software—can be transformed into cost-effective tools.

Third, to use digital media, there is a need for further research on software that can be combined with art therapy, and education in various application programs. Moreover, since few apps have been developed for use in art therapy, convergence research and development between interdisciplinary studies are necessary.

Fourth, digital art therapy and traditional art therapy are complementary to each other. Traditional and digital media have different characteristics in nature or expressive characteristics (11). Therefore, it is necessary for practitioners to have an in-depth understanding of both characteristics and differences between traditional and digital media. The author would like to suggest the importance of a complementary use of them for therapeutic purposes and for assisting in art therapy activities.

## 6. Conclusion

This study analyzed experiences and therapeutic meanings of adolescents with intellectual disabilities in group art therapy using digital media to derive six themes: (1) New and fun digital art therapy; (2) enjoying daily choices as the protagonist of life; (3) Discovery of the positive power and possibilities; (4) Digital art therapy enriching experience; (5) Preciousness, and joyfulness of the process of “being together”; and (6) Sublimation to digital art through immersion. Therapeutic factors that these themes imply in digital media have been identified as playfulness, interactionality, and scalability. The results of this study show that the use of digital media provides unlimited possibilities for participants to freely express their emotions in new ways and can improve various sensory experiences and creativity. This was an opportunity to develop positive relationships, improve self-esteem, and develop digital literacy skills by amplifying emotions of teenagers with intellectual disabilities and by changing their thinking, feelings, and acting in the process of communicating with the media. This qualitative case study provides implications that digital media can be a beneficial therapeutic intervention as it can provide everyone with more opportunities, including those with disabilities.

## Data availability statement

The original contributions presented in the study are included in the article/Supplementary material, further inquiries can be directed to the corresponding author.

## Ethics statement

Ethical review and approval was not required for the study on human participants in accordance with the local legislation and institutional requirements. Written informed consent to participate in this study was provided by the participants’ legal guardian/next of kin.

## Author contributions

JK and YC performed the conception and design this study. JK wrote the manuscript. YC critically reviewed the manuscript. All authors contributed to the article and approved the submitted version.

## Conflict of interest

The authors declare that this research was conducted in the absence of any commercial or financial relationships that could be construed as a potential conflict of interest.

## Publisher’s note

All claims expressed in this article are solely those of the authors and do not necessarily represent those of their affiliated organizations, or those of the publisher, the editors and the reviewers. Any product that may be evaluated in this article, or claim that may be made by its manufacturer, is not guaranteed or endorsed by the publisher.
